# Trimeric superoxide dismutase 1 antibody as a universal biomarker for ALS

**DOI:** 10.21203/rs.3.rs-6941118/v1

**Published:** 2025-07-15

**Authors:** Nikolay Dokholyan, Brianna Hnath, Rachel Dokholyan, Zachary Simmons

**Affiliations:** Penn State College of Medicine; Penn State College of Medicine; Penn State Health; Penn State Health

## Abstract

Amyotrophic lateral sclerosis (ALS) is a fatal neurodegenerative disease that leads to the loss of motor neurons, resulting in paralysis and death. Currently, there are no specific biomarkers available for diagnosing ALS. As a result, diagnosis currently relies on excluding other conditions, which forces patients to endure months or even years of uncertainty. The absence of a specific, reliable diagnostic tool has hindered both early intervention and therapeutic progress. Here we develop a novel synthetic antibody that can detect a toxic form of a known protein linked to ALS. This trimeric assembly of superoxide dismutase 1 (SOD1) is a soluble, structurally distinct oligomer that is highly toxic in cell models. The antibody selectively binds this trimer and differentiates individuals with the disease from healthy people and from those with other neurodegenerative diseases (Alzheimer’s and Parkinson’s disease). This breakthrough provides the first disease-specific diagnostic tool for this condition and reveals a shared pathological signature across patients, even in cases without genetic mutations. After decades without a specific diagnostic tool, this antibody signifies a long-awaited breakthrough, finally offering clinicians and researchers a reliable window into ALS pathology.

## Introduction

Over 5,000 people are diagnosed with amyotrophic lateral sclerosis (ALS) in the U.S. each year, with only 10% of cases being familial (fALS) and the remaining 90% classified as sporadic (sALS)^1,2^. Similar misfolded proteins are found in both forms, and since 1993, mutations in over 50 different genes have been linked to ALS, with more still being identified^3^. This genetic diversity has led researchers to debate whether ALS represents a single disease mechanism triggered by multiple factors (convergent) or a group of distinct diseases with similar symptoms (divergent)^4,5^. Developing a biomarker capable of diagnosing ALS across different etiologies (fALS and sALS) is challenging due to the limited understanding of the disease’s origins. Currently, there are no specific biomarkers for ALS; ongoing research focuses on broad neurodegeneration markers (such as p75ECD, phosphorylated neurofilament heavy chain, and neurofilament light) or inflammatory cytokines that require complex combinations to distinguish ALS patient samples^6,7^. The diagnosis of ALS is essentially a clinical one, relying on a combination of symptoms, signs, and electrodiagnostic findings that support the diagnosis, accompanied by blood and imaging studies (and sometimes studies of cerebrospinal fluid) to exclude diagnoses with similar phenotypes. Over time, various criteria have been published to optimize the sensitivity and specificity, such as El Escorial, Awaji, and Gold Coast Criteria^8–10^. These have generally been used to define inclusion criteria for clinical trial participation, and have some utility for diagnosis in clinical practice. However, all three criteria methods have difficulty with either specificity (leading to overdiagnosis) or sensitivity (missing early or complex cases). There remains a need for sensitive, specific, and objective biomarkers for diagnosis, monitoring of disease progression, and assessing response to treatment. The lack of a definitive biomarker delays diagnosis, often until substantial motor neuron loss has already occurred, at which point therapeutic options are limited in their ability to alter disease trajectory^11^. These challenges in current diagnostic strategies highlight the urgent need for a disease-specific biomarker that can reliably diagnose ALS and guide clinical decision-making.

The first gene linked to ALS was SOD1, which encodes a cytosolic antioxidant enzyme that normally functions as a copper- and zinc-bound dimer to convert superoxide radicals into oxygen and hydrogen peroxide^12^. Mutations in SOD1 destabilize the dimer interface, causing metal loss and promoting aggregation into soluble oligomers and insoluble fibrils^13,14^. Mutant SOD1 (G93A) mouse models develop a loss of motor function, while SOD1 knockdown mouse models do not display the same phenotype. This study led to the conclusion that SOD1 misfolding causes a toxic gain-of-function^15,16^. Although mutations in SOD1 are only found in 1–3% of all ALS patients, misfolded SOD1 has been found throughout patients with other ALS-related mutations (such as fused in sarcoma (FUS) and TDP-43) and in many sporadic cases^17–20^. Many other factors besides mutations can cause SOD1 to destabilize and aggregate, including post-translational modifications (e.g., glutathionylation)^21–23^, loss of metals^14,24^, crowding from overexpression^12^, and environmental toxins (such as BMAA and ammonia)^25–27^. Whether misfolded SOD1 is present in sporadic and non-SOD1 familial ALS patients has been highly disputed over the past decade, most likely due to inconsistencies in the antibodies used^19,28,29^, as well as different tissue types and preparations. Forsberg, Pare, Grad, and Pokrishevsky all observed misfolded SOD1 in non-SOD1 fALS and sALS patient spinal cord sections using either immunohistochemistry or immunoprecipitation^19,20,30,31^. In contrast, Da Cruz and Liu did not identify differences in misfolded SOD1 between ALS patients and controls; however, Da Cruz used very low concentrations of antibody, and Liu tested using only one monoclonal SOD1 antibody^32,33^. Collectively, this evidence suggests that misfolded SOD1 may play a central role in ALS pathology, even beyond cases with SOD1 mutations.

When the SOD1 dimer interface is disrupted, SOD1 aggregates and forms a wide range of soluble oligomers and insoluble fibrils^14,23,24,34^. Most previous studies consider misfolded SOD1 to mean a mix of all aggregate sizes; however, the different-sized oligomers exhibit drastically different toxicities and structures. In 2016, Proctor et al. determined that small soluble trimeric oligomers of SOD1 had the highest toxicity in cell models and that the toxicity correlated with the amount of thermodynamic stability of trimeric SOD1^35^. Zhu et al expanded on the toxicity of trimers by demonstrating that larger SOD1 oligomers and insoluble fibrils are protective to cells^36^. In 2022, Hnath and Dokholyan determined that trimeric SOD1 is a structurally independent species that forms in direct competition with larger aggregates, as opposed to trimeric SOD1 being a preliminary step in larger aggregate formation^37^. Due to aggregate structural differences, very few anti-misfolded SOD1 antibodies bind to trimeric SOD1; antiC4F6 is the only antibody we have identified that can consistently recognize trimeric SOD1^28,34,35^. The inability of most antibodies to detect trimeric SOD1 may account for the drastic inconsistencies between studies quantifying misfolded SOD1 in patient samples. We propose that trimeric SOD1 has an expansive role in ALS that has been hidden due to limitations in antibody specificity.

We developed the first antibody that specifically recognizes trimeric SOD1, a toxic and structurally distinct species that existing antibodies fail to detect with precision. Most commercially available misfolded SOD1 antibodies were not designed to distinguish between aggregate forms and often bind a mixture of oligomers and fibrils, limiting their diagnostic utility. To overcome this, we used a phage display-based synthetic design platform that allowed us to screen for antibodies with high specificity for trimeric SOD1 and minimal cross-reactivity to dimeric or fibrillar forms. When tested with patient serum, our novel antibody reliably detected trimeric SOD1 in sporadic ALS patients, not just those with known SOD1 mutations. These results establish trimeric SOD1 as a specific diagnostic biomarker for ALS and provide strong molecular evidence supporting a shared disease mechanism across genetically diverse ALS subtypes.

## Results

### ALS patients develop antibodies specifically against trimeric SOD1

**Figure 1:**
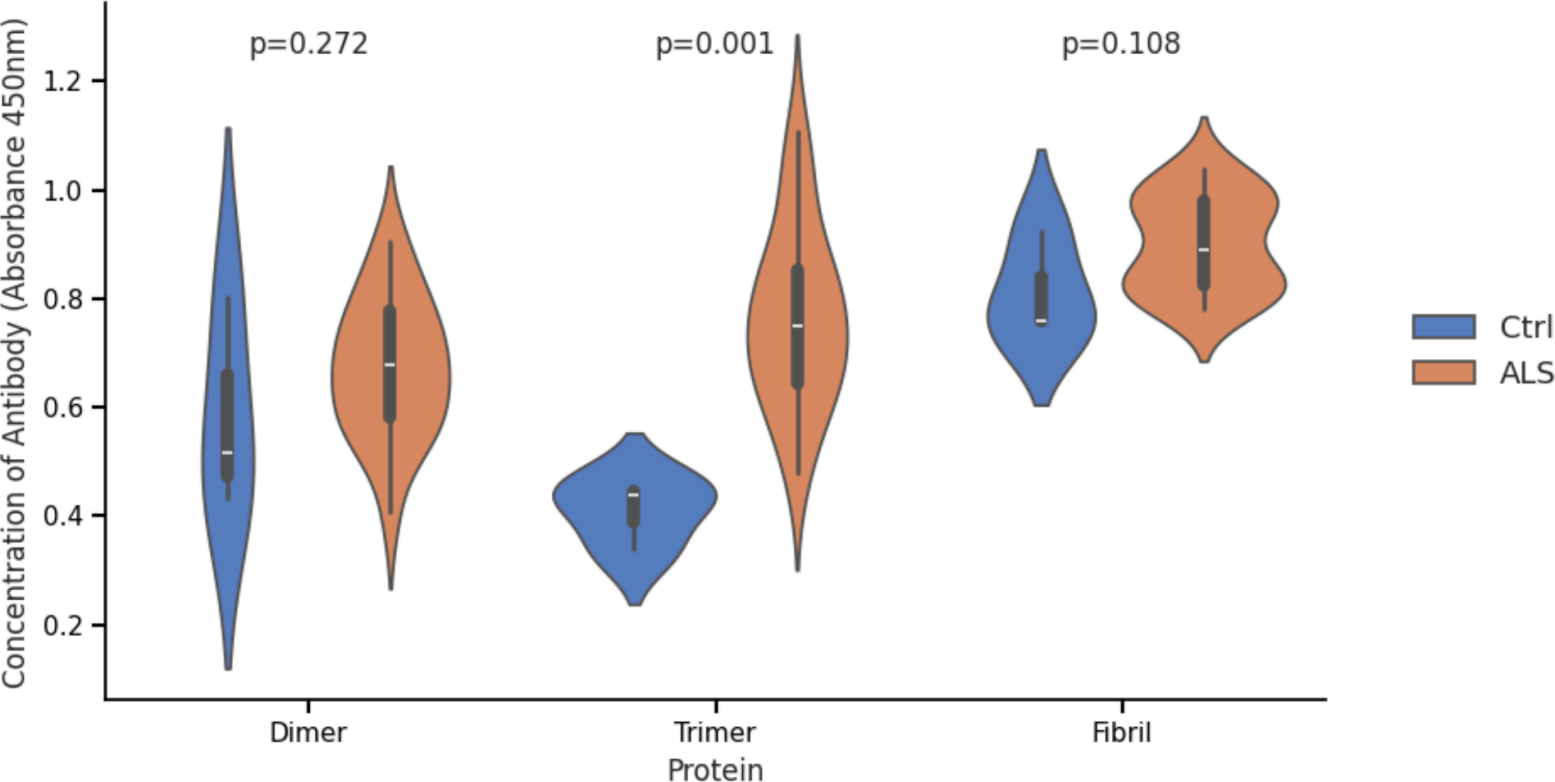
There is a significant increase in antibodies against trimeric SOD1 in ALS patient samples versus healthy controls.

To determine whether trimeric SOD1 is present in ALS patient samples we screened serum from 20 ALS patients (Table 1, Archived ALS samples) and three healthy controls for endogenous antibodies targeting structurally distinct SOD1 conformations: wild-type dimeric SOD1 (WT), stabilized trimeric SOD1 (F20L-H46Q), and large insoluble SOD1 fibrils (A4V) (Fig. 1)(His-tag on the purified trimer had no significant effect on the binding of patient antibodies, and whole blood and serum show no significant difference, Figure S1 & S2)^37^. Prior studies investigating misfolded SOD1 in ALS primarily relied on immunohistochemistry of spinal cord sections^19,20,29,31,32^, but this approach suffers from two major limitations: first, commonly used antibodies against misfolded SOD1 are inconsistent and often fail to detect all misfolded forms^28^; second, the high antibody concentrations required for staining make large-scale screening impractical^19^. To bypass these limitations, we used conformation-stabilizing SOD1 mutations to isolate and test specific aggregate forms. Our results revealed a significant increase in anti-trimeric SOD1 antibodies in ALS patients compared to healthy controls (p = 0.001), while responses to dimeric and fibrillar SOD1 showed no significant differences between groups (Fig. 1). The presence of antibodies against dimeric and fibrillar forms in both groups is likely explained by structural overlap with extracellular SOD3. These findings provide the first clear evidence that specifically toxic trimeric SOD1 is present in ALS patients without SOD1 mutations.

### Designing a synthetic antibody for trimeric SOD1

**Figure 2:**
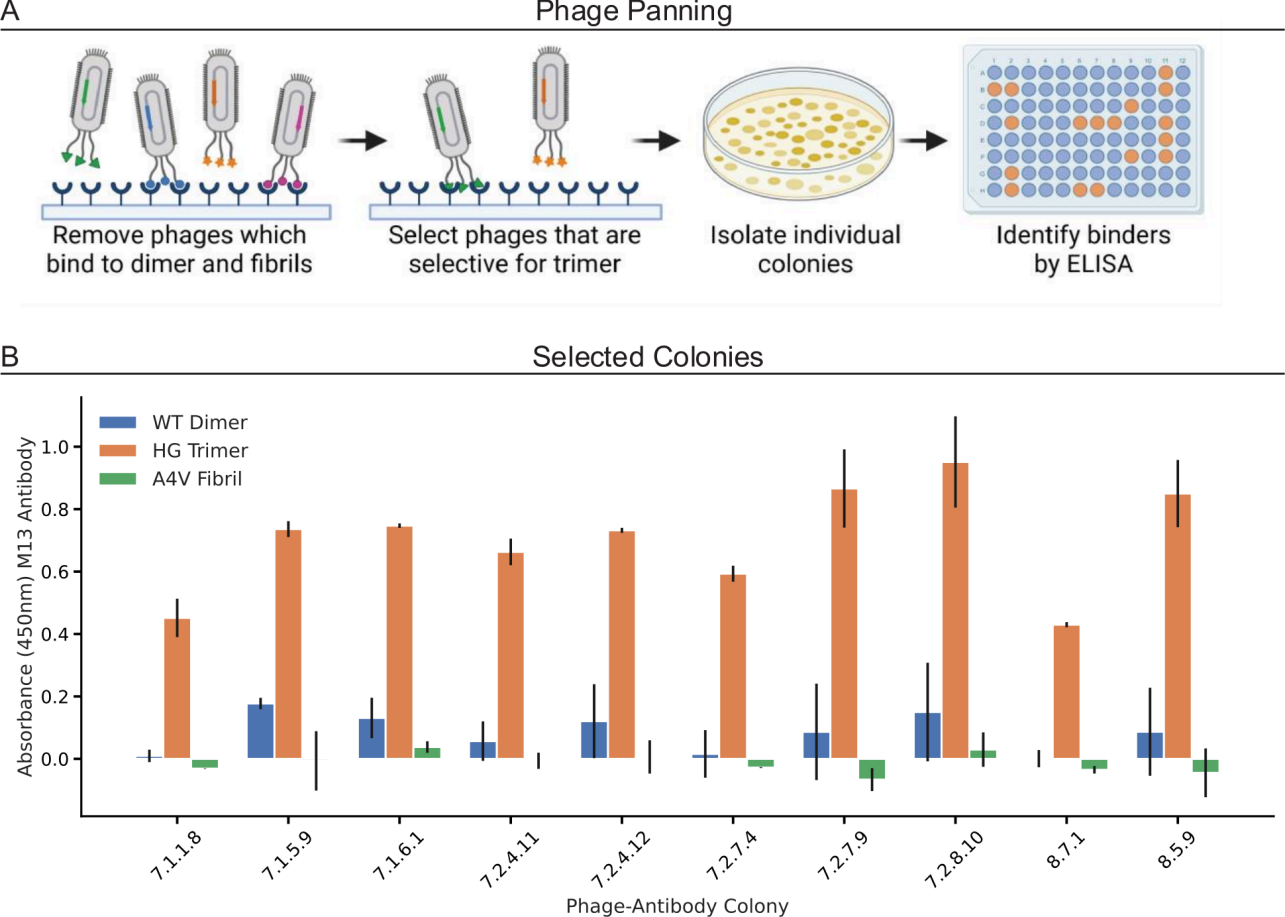
A) Schematic of phage panning to isolate potential trimer binding antibody “hits”. B) ELISA of original eight antibody “hits”, performed in technical triplicates.

**Figure 3:**
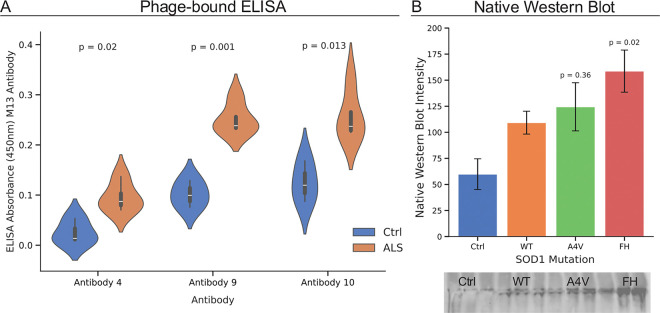
A) First ELISA serum test (five healthy controls and five ALS patients) of the three narrowed-down antibody “hits”. Antibody 9 is selected for additional optimization. B) Native-PAGE blot using antibody 9 to detect trimer in NSC-34 (motor neuron-like) cell lysate overexpressing different mutants of SOD1 that incrementally stabilize trimeric SOD1.

We utilized a phagemid synthetic human Fab antibody library to isolate candidate antibodies that specifically bind to trimeric SOD1 (Fig. 2A). Eight potential antibody “hits” were identified after seven rounds of phage panning. These initial “hits” exhibit binding to cell lysate with trimeric SOD1 overexpression (F20L-H46Q (FH) or H46Q-G108H (HG) trimer stabilizing mutations) but show no binding to control cell lysate or purified WT dimeric SOD1 (Fig. 2B). By assessing binding to cell lysate in the first stage, we removed any antibodies with non-specific binding to other common proteins. We narrowed the initial “hits” down to three based on native western blots: 7.2.7.4 (4), 7.1.5.9 (9), and 7.2.2.10 (10) (Supplementary Figure S3). We then refined the final three “hits” to “hit” 9 through an initial ELISA comparing the signal from the serum of five healthy control patients and five ALS patients. Antibody 9 demonstrated the most significant (p-value =0.001) difference between the healthy control and ALS groups in the initial blood testing (Fig. 3A).

### Trimeric SOD1 antibody as a biomarker assay for ALS

Using antibody 9 with the phagemid still attached causes additional noise when testing patient samples and leads to the antibody aggregating during storage, which reduces its activity (Supplementary Figure S4). To overcome these challenges, we created a shortened version of the DNA for antibody 9 with the M13 localization region removed. We expressed the human Fab protein in BL21 cells and purified it using a His column and size exclusion chromatography (SEC) (Supplementary Figure S5). Since antibody 9 is a human antibody, and our samples are also human, we crosslinked activated peroxidase directly to the purified antibody to avoid cross-reactivity from using an anti-human secondary antibody. The purified antibody 9 detected increasing levels of trimeric SOD1 in a native western blot as different SOD1 mutations incrementally stabilize the trimer in a motor neuron-like cell line (NSC-34) (Fig. 3B). The purified antibody also showed increased specificity in human patient samples compared to the same antibody with the phage still attached (Figure S5). Using surface plasmon resonance (SPR), we determined the binding affinity of antibody 9 with purified trimeric SOD1 (FH-His) compared to WT SOD1. Our novel antibody has strong binding (K_D_ of 7.92 × 10^−9^ M (± 1.01 × 10^−9^)) with trimeric SOD1; in comparison, we were unable to determine the binding affinity for the interaction with dimeric WT SOD1 due to a lack of signal (Supplementary Figures S6 & S7).

**Figure 4:**
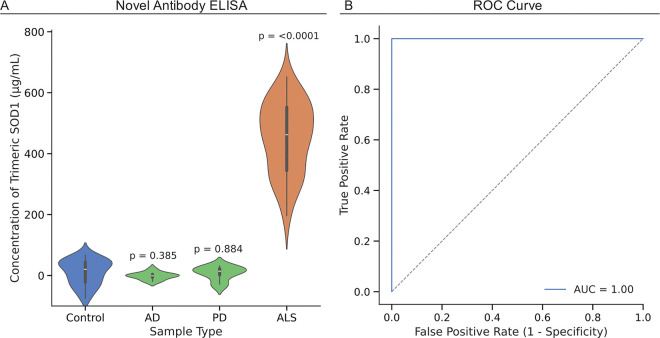
A) ELISA quantification of trimeric SOD1 in healthy control, Alzheimer’s Disease (AD), Parkinson’s Disease (PD), or ALS patient serum samples using antibody 9 at a 1:100 dilution (working concentration 1.6 μg/mL). B) ROC curve illustrating the diagnostic performance of trimeric SOD1 in distinguishing ALS from control samples, with an AUC of 1.00 and an optimal threshold of 196.58 μg/mL based on Youden’s J statistic.

To further determine if our new antibody for trimeric SOD1 is specific to ALS, we compared binding in serum samples from 42 healthy control patients, ten Alzheimer’s disease (AD) control patients, ten Parkinson’s disease (PD) control patients, and 42 newly collected ALS patient blood samples (collected between October 2024 and May 2025) (Table 1). We observed a significant increase in trimer levels in the ALS patients compared to the healthy control group (p < 0.0001), but no difference between the healthy control and Alzheimer’s disease patients or Parkinson’s disease patients (AD p = 0.385, PD p = 0.884)(Fig. 4A). A receiver operating characteristic (ROC) curve was generated to evaluate the diagnostic performance of trimeric SOD1 concentrations in distinguishing ALS from control samples. The analysis yielded an area under the curve (AUC) of 1.00, indicating perfect discrimination in this dataset (Fig. 4B). The optimal threshold, determined using Youden’s J statistic, was identified at 196.58 μg/mL, with both sensitivity and specificity reaching 100%. This threshold reflects the point of maximal separation between ALS and all control groups. Titer estimations for trimeric SOD1 were performed using serial dilutions ranging from 1 μg/mL to 100 μg/mL to conserve protein material. While this range provided sufficient dynamic coverage for the control, Alzheimer’s disease, and Parkinson’s disease samples, some ALS samples exhibited saturation or underestimated titers due to concentrations falling above the upper limit of the curve. As a result, ALS sample titers may be slightly underestimated, though this does not appear to affect the overall discriminatory capacity of the biomarker as demonstrated by the ROC analysis.

## Discussion

We identified and validated trimeric SOD1 as a highly specific and disease-relevant biomarker for ALS using a newly developed synthetic antibody. This antibody was specifically designed to recognize the structurally distinct trimeric form of SOD1 and demonstrated high specificity in both direct ELISA and Western blot assays. Using this tool, we consistently detected trimeric SOD1 in serum samples from ALS patients, primarily those with sporadic disease, but not in samples from healthy controls or individuals with other neurodegenerative conditions. Additionally, we found that ALS patients produced endogenous antibodies against trimeric SOD1, further confirming its presence in circulation. These findings establish trimeric SOD1 as a distinct pathological species and a viable target for blood-based detection.

The ability to detect trimeric SOD1 in serum provides a practical advantage over many existing ALS biomarkers, which often rely on cerebrospinal fluid or muscle biopsy samples^39^. Obtaining these sample types is more invasive and less accessible for longitudinal studies^40,41^. Some potential new biomarkers can be detected in blood samples. Two of the most commonly used biomarkers currently are neurofilament light chain (NfL) and phosphorylated neurofilament heavy chain (pNfH), which can be assayed in both blood and CSF. As per a recent systematic review and meta-analysis: “Both higher levels of NfL and pNfH either measured in blood or CSF were correlated with more severe symptoms as assessed by the ALS Functional Rating Scale Revised score and with a faster disease progression rate; however, only blood NfL levels were associated with shorter survival.”^42^. However, NfL and pNfH do not help to understand ALS pathogenesis, and both (especially NfL) are found elevated in other neurological conditions, hindering their usefulness as diagnostic biomarkers^42^. The synthetic antibody developed in this study addresses longstanding challenges related to antibody inconsistency and heterogeneity among SOD1 aggregates. Previous studies that aimed to detect misfolded SOD1 as a biomarker reported elevated anti-SOD1 antibody levels in sporadic ALS patients, and these levels sometimes correlated with disease severity^43^. However, inconsistent antibody specificity and the inability to resolve distinct conformations within mixed aggregate populations limited the usefulness of those findings. Our approach addresses these limitations by targeting a single defined and toxic species, trimeric SOD1, using recombinant protein testing to confirm specificity. Additional evidence supports the extracellular presence of misfolded SOD1, including trimeric forms^44^. Misfolded SOD1 has been found on the surface of extracellular vesicles secreted by ALS cell models (HEK293, Neuro2a, and NSC-34)^45–47^ as well as in ALS mouse models^46,48^. Our findings are consistent with these observations and suggest that trimeric SOD1 may contribute to disease propagation through extracellular mechanisms. The ability to detect trimeric SOD1 directly in serum highlights its promise as a minimally invasive and scalable biomarker for ALS.

Despite its potential, there are still technical challenges to the use of trimeric SOD1 in diagnostic assays, which we have overcome by developing a trimer-specific antibody. Trimeric SOD1 is structurally fragile and degrades after repeated freeze and thaw cycles. We observed this effect in our initial cohort, which included samples collected between 2014 and 2020 that had undergone multiple freeze and thaw cycles (Table 1, Archived ALS Samples). These archived samples, although suitable for antibody detection due to the stability of immunoglobulins, showed significantly reduced trimeric SOD1 signal compared to samples collected more recently (Supplementary Figure S8). An assay directly quantifying patient immunoglobulin levels is not scalable for widespread diagnostic use because it requires large amounts of highly purified trimeric SOD1. Generating this conformer involves inducing aggregation over several days and isolating only the trimeric fraction, which requires multiple purification steps and results in low yield. These limitations are addressed by the synthetic antibody developed in this study. The direct detection assay we established (utilizing our novel trimeric SOD1 antibody) requires only a small amount of purified trimeric SOD1 to generate a standard curve. The antibody itself can be produced at scale using established expression and purification protocols^49^, and allows for consistent and reproducible detection. Our results also indicate that trimeric SOD1 remains stable with limited freeze and thaw cycles, and that sample integrity can be preserved by minimizing handling or using freshly collected samples.

A reliable biomarker is essential for accurate diagnosis and for consistent monitoring of ALS progression^40,41^. The large effect size and strong statistical significance observed between ALS patients and both healthy and neurological disease controls support the diagnostic potential of trimeric SOD1. Moreover, we identified a concentration threshold that strongly differentiates ALS cases from non-ALS controls (196.58 μg/mL), representing a key step toward clinical implementation. These results also have broader implications for how we understand ALS as a disease. The detection of trimeric SOD1 in sporadic ALS patients supports the convergence model of ALS. Our finding that trimeric SOD1 is detectable in sporadic ALS patients suggests the presence of a common toxic species across diverse ALS cases. This supports a unified view of ALS in which different initiating events lead to a similar downstream molecular pathology^50^. Recognizing ALS as a convergent disease shifts the research and diagnostic focus toward identifying consistent molecular markers that reflect this shared pathology. A biomarker like trimeric SOD1, which is detectable across patients with different backgrounds, provides a foundation for more standardized diagnosis. The synthetic antibody described here not only enables this detection but also offers a powerful tool for further investigations into ALS pathogenesis.

In summary, we establish trimeric SOD1 as a highly specific and extracellular biomarker for ALS and present a synthetic antibody that enables its reliable detection in patient serum. These results support the convergence model of ALS and offer the first specific biomarker for diagnosis and future research of ALS.

## Methods

### SOD1 Protein Purification

The His-TEV WT SOD1 plasmid was ordered in a pET-28a(+)-TEV backbone from Genscript. Point mutations (A4V, F20L-H46Q, H46Q-G108H) were introduced via site-directed mutagenesis using primers from MilliporeSigma, and all mutated constructs were sequence-verified by Genewiz/Azenta. Proteins were expressed in *E. coli* grown in LB broth at 37 °C, with induction of expression using 1 mM IPTG at 18 °C. Cells were harvested by centrifugation at 7,000 × g for 30 minutes and resuspended in lysis buffer (20 nM sodium phosphate dibasic, 500 Mm NaCl, 20 mM imidazole, 1 mM PMSF, and 1 μM Pepstatin A). Lysis was performed using probe sonication. His-tagged SOD1 was purified from the lysate supernatant using a 5 mL HisTrap column on an AKTA Pure FPLC system and eluted with 500 mM imidazole. The eluted protein was concentrated using a spin concentrator and buffer-exchanged into 1X PBS. Aggregation was induced by incubating the protein at 37 °C for 72 hours, followed by purification of the resulting species using a 50 mL S200 size exclusion chromatography (SEC) column. For assays requiring removal of the His tag, TEV protease was added at a 1:100 ratio to total protein. Samples were incubated at 4 °C and passed through an additional SEC column to separate cleaved protein from free His tag. WT and A4V fibrils were generated by incubating protein at 37 °C for one week, followed by isolation of the insoluble fraction by centrifugation.

### Patient samples and genetics

Peripheral blood samples were collected from ALS clinic patients using K2EDTA tubes under Pennsylvania State University IRB protocol PRAMS #43763 under the leadership of Dr. Zachary Simmons (and used by Dr. Nikolay Dokholyan IRB protocol PRAMS #43763EP). Newly collected samples were centrifuged at 1300 × g for 20 minutes at room temperature to isolate plasma, which was then aliquoted and stored at −80°C until further analysis. Each freeze-thaw cycle was documented in a sample log to ensure consistency and maintain sample integrity. Sex, age, site of onset, and revised ALS functional rating scale (ALSFRS-R) score^38^ were obtained for each patient sample. Due to IRB constraints, individual genetic data for ALS patients could not be disclosed; however, population-level estimates from the ALS clinic providing the samples indicated that over 90% of the ALS cohort consisted of sporadic cases, consistent with known epidemiological patterns. An initial pilot study comparing ALS (n = 20) and control (n = 3) plasma samples demonstrated a statistically significant difference (*p* = 0.0014) with a very large effect size (Cohen’s *d* = 2.28), the ALS samples used in this pilot study were collected between 2014–2020 and underwent an unknown number of freeze/thaw cycles. Based on this strong effect size, a power analysis indicated that a minimum of 26 samples per group would be sufficient to detect a significant difference with 80% power at α = 0.05. To exceed this threshold and enhance robustness, 42 samples were ultimately included for both the ALS and healthy control groups. We obtained age- and sex-matched, healthy control serum samples, free from known neurological disorders and negative for viral testing, as well as ten Alzheimer’s disease serum samples from the commercial supplier Precision for Medicine. Additionally, ten Parkinson’s disease serum samples were obtained from the Pennsylvania State University Translational Brain Research Center under the leadership of Dr. Xuemei Huang, IRB protocol #40726.

### Patient Antibody ELISA

Purified SOD1 proteins (WT, F20L-H46Q trimer, H46Q-G108H trimer, WT fibril, or A4V fibril) were diluted in 1X PBS to equivalent concentrations and coated onto Nunc Maxisorp ELISA plates (50 μL per well) overnight at 4 °C. Plates were washed three times with 1 X PBS and then blocked with 1% bovine serum albumin (BSA) overnight at 4 °C. Patient serum or whole blood samples were diluted 1:10 in 1X PBS and added to the blocked plates for 3 hours at 37 °C. After washing three times with 1X PBS, plates were incubated with anti-human HRP-conjugated secondary antibody (1:1,000 in 1% BSA) overnight at 4 °C. Plates were washed again and developed using 100 μL per well of TMB solution for 5 minutes, followed by the addition of 50 μL per well of 2 N sulfuric acid to stop the reaction. Absorbance was measured at 450 nm using a SpectraMax plate reader. To determine the relative antibody levels, a direct ELISA was performed using the same secondary antibody and 1:10 diluted patient samples. Absorbance values were background-subtracted (no patient sample control) and normalized to total antibody levels prior to graphing and statistical analysis in Python.

### Synthetic Antibody Design

An M13 phagemid human Fab phage display library (2.0 × 10^9^ mutants) was obtained from Leading Biology, along with M13K07 helper phage. The helper phage was amplified using the vendor’s protocol. Briefly, growth-phase XL1-Blue *E. coli* was cultured in 2YT-tetracycline medium (Bacto tryptone, Bacto yeast extract, NaCl, and 10 μg/mL tetracycline) and infected with helper phage. Following an initial growth period (30 minutes without shaking, then two hours with shaking), kanamycin was added to a final concentration of 25 μg/mL. The culture was incubated with shaking, harvested by centrifugation, and the supernatant filtered through a 0.45 μm sterile filter to collect amplified helper phage. Phage titers were determined by plating serial dilutions on agar, and aliquots were stored in 7% DMSO at −80 °C.

The phage display library was amplified using a similar approach. XL1-Blue *E. coli* in mid-log phase was infected with the library and incubated at 37 °C. Ampicillin was added to a final concentration of 100 μg/mL, followed by incubation and infection with helper phage. The culture was centrifuged, resuspended in 2YT medium supplemented with ampicillin (100 μg/mL), tetracycline (10 μg/mL), and kanamycin (50 μg/mL), and incubated with shaking. After centrifugation, the phage-containing supernatant was mixed with one-fifth volume of PEG-NaCl buffer (20% PEG 6000, 2.5 M NaCl) to precipitate the phage. The mixture was incubated on ice, pelleted by centrifugation at 10,000 × g for 25 minutes at 4 °C, and the pellet was resuspended in 1 mL 1X PBS containing 1% BSA. Phage titers were again determined before use in panning. To enrich for trimer-specific binders, conformation-stabilized SOD1 proteins–dimeric WT, trimeric F20L/H46Q, trimeric H46Q/G108H, and insoluble aggregates of A4V or WT–were coated at 10 μg/mL in 1X PBS on ELISA plates. After washing and blocking with 3% BSA, 100 μL of the concentrated phage library was first incubated on WT dimer-coated wells to remove non-specific binders. The phage solution was then transferred sequentially to wells coated with A4V or WT aggregates, followed by final transfer to the trimer-coated wells (F20L/H46Q or H46Q/G108H). After binding, wells were washed eight times with 1X PBS to remove unbound phage, and bound phage was eluted using 0.2 M glycine (pH 2.2). The eluate was neutralized with 1 M Tris (pH 9.0), then added to log-phase XL1-Blue *E. coli*. The phage library was re-amplified as described above. This panning process was repeated for seven rounds (Supplementary Table S1), with phage titers and library samples collected after each round.

During the seventh round, colonies were isolated by plating the enriched library on 2YT-ampicillin-tetracycline agar plates. Individual colonies were grown in 96-well plates and expanded. Supernatants containing the secreted phage were added to ELISA plates coated with either control NSC-34 cell lysate, trimer-overexpressing NSC-34 lysate, or purified WT dimeric SOD1. Plates were blocked with 1% BSA, incubated with anti-M13 HRP-conjugated antibody (1:1000 in 1% BSA), and developed using TMB substrate and sulfuric acid. Absorbance was measured using a SpectraMax plate reader. Colonies were considered hits if they produced a signal above 0.05 (normalized to background) for trimer-overexpressing lysate but showed no signal for control lysate or WT dimer. The top eight hits were selected for secondary screening by ELISA against dimeric WT, H46Q/G108H trimer, and A4V aggregates, using technical triplicates and a higher phage concentration. These clones were also tested by native Western blot as primary detection reagents against NSC-34 lysates expressing control (C), WT (W), A4V (A), or F20L/H46Q (F) SOD1. Based on both ELISA and Western blot data, three clones were selected for further validation.

### Patient Serum ELISA

Patient samples were evaluated for trimeric SOD1 concentration using the newly developed antibodies, either with or without a bacteriophage tag, following a similar setup to the patient antibody ELISA. Samples from healthy controls, Alzheimer’s disease patients (AD), Parkinson’s disease patients (PD), and newly collected ALS patients were diluted 1:10 in 1X PBS and coated in duplicate on ELISA plates. A titration curve of purified trimeric SOD1 protein (H46Q-G108H with His tag, ranging from 1 μg/mL to 100 μg/mL) was also coated in duplicate on the same plates. After sample coating, plates were washed with 1X PBS and blocked with 1% BSA to prevent non-specific binding. The experimental antibody (either phage-displayed or HRP-conjugated) was applied to the blocked plates. For detection, M13-specific secondary antibody was used when phage-displayed antibodies were applied; no secondary was required when using HRP-tagged antibodies. Plates were developed using TMB substrate, and the reaction was stopped with sulfuric acid. Absorbance was measured at 450 nm using a SpectraMax plate reader. Background absorbance from uncoated wells was subtracted from each reading, and duplicate wells were averaged. Signals were compared to the trimeric SOD1 titration curve to estimate concentration. Statistical analysis, including two-sample *t*-tests, ROC curve generation, and calculation of Youden’s J statistic, was performed using Python.

### Antibody Purification and HRP Conjugation

The M13 phage region was removed from the isolated antibody plasmids by restriction digestion with *Spel* and *Nhel* (New England Biolabs), followed by ligation with T4 ligase and transformation into BL21 *E. coli*. The Fab antibody was expressed and purified using the same protocol as for SOD1 purification. To generate an HRP-conjugated Fab, 1 mg of purified antibody was incubated with 1 mg of lyophilized EZ-Link activated peroxidase in 10 mM sodium carbonate buffer. Crosslinking was initiated by adding 10 μL of sodium borohydride and incubating the mixture under gentle agitation. The reaction was quenched with 20 μL of 3 M ethanolamine. The peroxidase-conjugated Fab was then isolated by size exclusion chromatography (SEC).

### Surface Plasmon Resonance (SPR)

To quantify the binding affinity between the novel antibody and trimeric versus wild-type (WT) SOD1, we performed surface plasmon resonance (SPR) using a 2-channel Nicoya OpenSPR instrument equipped with a high-capacity carboxyl sensor. The antibody was immobilized on the sensor surface using Nicoya’s carboxyl coupling kit: carboxyl groups were activated with EDC/NHS, followed by injection of purified antibody (50 μg/mL in 10 mM HCl, pH3.0) until signal exceeded the recommended 45 RU threshold for 1:1 binding. Residual activated groups were quenched using 1M ethanolamine (pH 8.5). Purified dimeric (WT) or trimeric (FH-His) SOD1 was then injected at concentrations ranging from 57 pM to 75 nM in 1X PBS. Regeneration between injections was achieved using 10 mM glycine-HCl (pH 1.5). Data were analyzed in TraceDrawer. Due to inconsistencies in the dissociation phase across concentrations, kinetic fitting (k_a_, k_d_) was unreliable. Instead, steady-state response values from three antibody–antigen concentrations were used to estimate the equilibrium dissociation constant (K_D_) using a 1:1 binding model.

## Supplementary Material

Supplementary Files

This is a list of supplementary files associated with this preprint. Click to download.

• hdxxx25bSupplementary.docx

## Figures and Tables

**Table 1.** Clinical characteristics of patients with ALS and controls with no known neurological disorders, Alzheimer’s disease (AD), or Parkinson’s disease (PD).

Characteristic	Archived ALS Samples (2014–2020) (n=20)	ALS (2024–2024) (n=42)	Healthy Controls (n=42)	AD (n=10)	PD (n=10)
Male, n (%)	17 (85%)	27 (64.3%)	27 (64.3%)	5 (50%)	5 (50%)
Age, yr	62 (35–81)	66 (34–83)	64 (46–86)	76.8 (68–85)	(61–80)
Disease duration, months		29.3 (0–326)			
Site of onset (limb/bulbar)	9/4 (7 N/A)	23/12			
ALSFRS-R score	27.7 (6–48)	34.9 (20–47)			
No genetic mutation (%)		39 (92.9%)			
